# Hypoxanthine-guanine phosophoribosyltransferase (HPRT) deficiency: Lesch-Nyhan syndrome

**DOI:** 10.1186/1750-1172-2-48

**Published:** 2007-12-08

**Authors:** Rosa J Torres, Juan G Puig

**Affiliations:** 1 Division of Clinical Biochemistry, La Paz University Hospital, Madrid, Spain; 2 Division of Internal Medicine, La Paz University Hospital, Madrid, Spain

## Abstract

Deficiency of hypoxanthine-guanine phosphoribosyltransferase (HPRT) activity is an inborn error of purine metabolism associated with uric acid overproduction and a continuum spectrum of neurological manifestations depending on the degree of the enzymatic deficiency. The prevalence is estimated at 1/380,000 live births in Canada, and 1/235,000 live births in Spain. Uric acid overproduction is present inall HPRT-deficient patients and is associated with lithiasis and gout. Neurological manifestations include severe action dystonia, choreoathetosis, ballismus, cognitive and attention deficit, and self-injurious behaviour. The most severe forms are known as Lesch-Nyhan syndrome (patients are normal at birth and diagnosis can be accomplished when psychomotor delay becomes apparent). Partial HPRT-deficient patients present these symptoms with a different intensity, and in the least severe forms symptoms may be unapparent. Megaloblastic anaemia is also associated with the disease. Inheritance of HPRT deficiency is X-linked recessive, thus males are generally affected and heterozygous female are carriers (usually asymptomatic). Human HPRT is encoded by a single structural gene on the long arm of the X chromosome at Xq26. To date, more than 300 disease-associated mutations in the HPRT1 gene have been identified. The diagnosis is based on clinical and biochemical findings (hyperuricemia and hyperuricosuria associated with psychomotor delay), and enzymatic (HPRT activity determination in haemolysate, intact erythrocytes or fibroblasts) and molecular tests. Molecular diagnosis allows faster and more accurate carrier and prenatal diagnosis. Prenatal diagnosis can be performed with amniotic cells obtained by amniocentesis at about 15–18 weeks' gestation, or chorionic villus cells obtained at about 10–12 weeks' gestation. Uric acid overproduction can be managed by allopurinol treatment. Doses must be carefully adjusted to avoid xanthine lithiasis. The lack of precise understanding of the neurological dysfunction has precluded development of useful therapies. Spasticity, when present, and dystonia can be managed with benzodiazepines and gamma-aminobutyric acid inhibitors such as baclofen. Physical rehabilitation, including management of dysarthria and dysphagia, special devices to enable hand control, appropriate walking aids, and a programme of posture management to prevent deformities are recommended. Self-injurious behaviour must be managed by a combination of physical restraints, behavioural and pharmaceutical treatments.

## Disease name and synonyms

The deficiency of the enzymatic activity of hypoxanthine-guanine phosphoribosyltransferase (EC 2.4.2.8; HPRT) is associated with two OMIM items. Lesch-Nyhan syndrome (OMIM 300322) corresponds with virtually complete HPRT deficiency and was described by M. Lesch and W. Nyhan in 1964 [1]. In 1967 Seegmiller, Rosenbloom and Kelly reported a complete deficiency of HPRT activity as the cause of the Lesch-Nyhan syndrome [2]. This same year, Kelly, Greene, Rosenbloom, Henderson and Seegmiller described a partial deficiency of HPRT activity associated with gout and no neurological involvement [3,4]. This partial deficiency was termed Kelly-Seegmiller syndrome or HPRT-related gout (OMIM 300323). Nowadays it is considered that between both syndromes, a continuous spectrum of neurological involvement is present in HPRT-deficient patients. The term Lesch-Nyhan variants has been introduced to include patients with HPRT-related gout and some degree of neurological involvement, but without the complete Lesch-Nyhan syndrome.

In 1959, before the Lesch and Nyhan description, Catel and Schmidt described an 18-month old infant with hyperuricemia, hyperuricosuria and encephalopathy [5]. They termed this clinical syndrome as "Hyperuricemic encephalopathy". This patient was later shown to suffer HPRT deficiency.

## Definition and diagnostic criteria

HPRT deficiency is characterized by hyperuricemia with hyperuricosuria and a continuum spectrum of neurological manifestations, which depends on the severity of the defect. These manifestations include severe action dystonia, choreoathetosis, mild to moderate mental retardation, and self-mutilation in the complete form or Lesch-Nyhan syndrome [6], that can go unnoticed in the mildest forms [4].

The association of a psychomotor delay in the first year of life with hyperuricemia and/or elevated uric acid to creatinine ratio suggest the possibility of HPRT-deficiency. On the other side of the spectrum, a patient with juvenile gout and elevated urinary uric acid excretion may also suffer HPRT deficiency.

## Epidemiology

HPRT deficiency is inherited as a recessive X-linked trait [7]. Thus, males are generally affected and women are generally asymptomatic carriers. At least five women with Lesch-Nyhan syndrome due to a variety of molecular mechanisms have been described in the literature [8-15]. The prevalence of the disease is estimated to be 1/380,000 live births in Canada [16], and 1/235,000 live births in Spain.

## Clinical description

Patients with HPRT deficiency are normal at birth. One of the first signs of the disease may be the observation of orange crystals in the diapers, or crystalluria with obstruction of the urinary tract. Other uncommon forms of presentation include renal failure or acidosis with repeated vomiting. Psychomotor delay, when present, becomes evident within 3 to 6 months. A delay in the acquisition of sitting and head support with hypotonia and athetoid movements may lead to neurological consultation. Self-mutilation, in the form of lip biting or finger chewing, can appear as soon as teeth are present [17,18].

Clinical features of HPRT deficiency include: uric acid overproduction-related symptoms, neurological manifestations, and haematological disturbances.

### Hyperuricemia-related renal and articular symptoms

These symptoms are present in all HPRT-deficient patients and are not related to the severity of the enzyme defect. All characteristic findings associated with gout may be present (acute arthritis, tophi, nephrolithiasis or urolithiasis, and renal disease). Hyperuricemia-related presentations include orange crystals in the diapers, crystalluria in the first years of life, or juvenile arthritis. If the diagnosis and treatment is delayed, tophi and renal failure may appear. However, nowadays allopurinol treatment prevents the development of gouty manifestations.

### Neurological symptoms

Neurological symptoms may differentiate HPRT-deficient variants. The presence and severity of these symptoms are relevant for prognosis. They may reflect the degree of enzyme deficiency and can render the patient partially or completely dependent on others for daily activities and personal care. We have proposed a classification of HPRT deficiency in four grades according to the severity of the neurological symptoms (see below) [18]. Other classifications in three grades have also been proposed [19]. Neurological symptoms affect the motor sphere, cognitive, and behavioural aspects.

◦ *Motor disorder: *In their original report on two affected brothers, M. Lesch and W. Nyhan [1] described the motor abnormalities as spasticity and choreoathetosis. However, in a recent revision by H. A. Jinnah *et al*. [17], the motor syndrome of complete HPRT deficiency is best classified as a severe action dystonia, superimposed on a baseline hypotonia. Dystonia is generalized to all parts of the body and its severity may lead to an inability to stand up and walk, and patients are confined to a wheelchair. Involuntary movements such as choreoathetosis and ballismus are usually present but are not evident at rest. These symptoms are associated with voluntary movements and increase with excitement and anxiety. Dysarthria and dysphagia and opisthotonus are frequently reported. Corticospinal tract signs such as spasticity, hyperreflexia and extensor plantar reflex are generally reported in later years and they may reflect an acquired defect.

In partial HPRT-deficient patients, a complete motor syndrome may be present in the most severe forms. In other patients the grade of dystonia is less severe and appears in the form of a dystonic gait, speech difficulties, exercised-induced dystonia or is unapparent.

◦ *Cognitive impairment*: The first Lesch-Nyhan description included mental retardation as a characteristic of the syndrome. However complete HPRT-deficient patients, when evaluated with specific tests for motor difficulties, show mild to moderate mental retardation. These patients show attention deficit disorders but the non-verbal intelligence is well preserved in the majority [20-22].

Partial HPRT deficient patients can present variable degrees of mental retardation including apparently normal intelligence, but they usually show various grades of attention deficit [20].

◦ *Compulsive self-injurious behaviour *is the most striking feature of Lesch-Nyhan syndrome and is only present in patients with the complete enzyme defect, although some Lesch-Nyhan patients never show auto-destructive behaviour. The patients begin to bite their lips, tongue or fingers and, without restrictions, important auto-mutilating lesions can develop [23,24]. The mutilation is not the result of a lack of sensation (the patients feel pain and are relieved when protected from themselves) and recently it has been ascribed to an obsessive-compulsive behaviour. In some instances, the aggressive behaviour is also directed against family and friends, with patients spitting or using abusive language. Self-mutilation may start between 2 and 16 years of age and, in some instances, is associated with or aggravated by psychological stress (adolescence, familial conflicts). Despite their periodic aggressive behaviour, Lesch-Nyhan patients are frequently happy and engaging children when they are restrained. The neuro-behavioural disorder may be markedly modulated by numerous environmental factors, among which education is the most relevant [25]. Partial patients do not show self-injurious behaviour but in some cases an obsessive-compulsive disorder has been reported.

### Haematological aspects

In Lesch-Nyhan patients the presence of megaloblastic anaemia is frequent and in some cases severe [26]. Microcitic anaemia can also be present, usually associated with hiatus hernia.

## Classifications

HPRT deficiency can be classified according to the severity of the neurological manifestations and the enzyme defect. The first classification includes HPRT-deficient patients as complete or Lesch-Nyhan syndrome, and as partial or Kelly-Seegmiller syndrome [3]. Other classifications include three groups: classical Lesch-Nyhan or complete deficiency (LN); HPRT deficiency with neurological manifestations or HPRT related hyperuricemia with neurological disability (HRND), and HPRT-related hyperuricemia (HRH) for partial patients with no evident neurological manifestations [17]. We have proposed a classification (Table 1) with four groups based on the clinical, biochemical, enzymatic and molecular data, derived from careful clinical observation of 22 patients from 18 different Spanish families [18]. We have divided the HRND group into two (Group 2 and 3) because neurological disability in these patients may vary between severe neurological symptoms and mild neurological symptoms with various prognoses.

**Table 1:** Classification of HPRT deficiency based on clinical, biochemical, enzymatic and molecular data.

	Partial deficiency (Kelly-Seegmiller syndrome)			Lesch-Nyhan
	Grade 1	Grade 2	Grade 3	Grade 4
Patients	6	3	2	25
HPRT haemolysate	(+)	(-)	(-)	(-)
HPRT erythrocyte	(+)	(+)	(-)	(-)
Size of protein altered	(-)	(-)	(-)	(+,-)
Self-mutilation	(-)	(-)	(-)	(+)

We have proposed a classification in four groups based on the clinical, biochemical, enzymatic and molecular data, derived from careful clinical observation of 36 patients from different Spanish families [18]. (+) Present or detectable. (-) Absent or undetectable. (+ -) May be present or not.

- *Group 1: normal development without neurological symptoms*. HPRT deficiency in these patients could be manifested as asymptomatic hyperuricemia with elevated uric acid excretion rates, or as renal lithiasis and/or gout. These patients are totally independent in terms of daily activities and lead normal lives. Only when carefully examined, they may present some minor dystonia, such as exercise-induce dystonia, attention deficit or obsessive-compulsive behaviour.

- *Group 2: mild neurological symptoms*. These patients have mild neurological symptoms such as dystonic gait, dysarthria, stuttering, and some degree of mental retardation. They are hampered by their neurological symptoms, although they are independent in most activities, and can walk and live on their own.

- *Group 3: severe neurological symptoms*. Patients are mentally normal, can feed themselves and take care of some of their personal needs, but their severe dystonia confines them to a wheelchair. These patients do not present self-injurious behaviour.

- *Group 4: classic Lesch-Nyhan syndrome*. These patients exhibit typical characteristics of Lesch-Nyhan syndrome, including self-injurious behaviour, choreoathetosis and ballismus, inability to stand or walk, some degree of spasticity, and are fully dependent on others for daily activities and personal needs. Some of these patients may not show auto-destructive behaviour or mental retardation.

This classification has been useful for the 36 HPRT deficient patients that we have diagnosed for the last 23 years (1984–2007). However, use of this classification system may require some time for the disease to be fully recognized (*i.e. *grade 2 and 3 can only be differentiated after 1 year of age).

## Aetiology

### Uric acid overproduction

**Figure 1:**
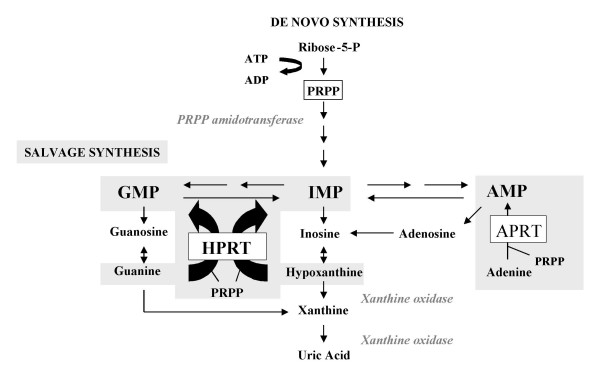
**Purine metabolism**. The metabolic scheme shows the first and rate-limiting step of *de novo *purine synthesis mediated by the enzyme 5'-phosphoribosyl-1-pyrophosphate (PRPP) amidotransferase, and the salvage pathway mediated by hypoxanthine phosphorybosyltransferase (HPRT) and adenine phosphorybosyltransferase (APRT). The *de novo *synthesis occurs through a multi-step process and requires the contribution of four aminoacids, one PRPP, two folates and three ATP to synthesize an inosine monophosphate (IMP) molecule. HPRT catalyzes the salvage synthesis of inosine monophosphate (IMP) and guanosine monophosphate (GMP) from the purine bases hypoxanthine and guanine respectively, utilizing PRPP as a co-substrate. The HPRT defect results in the accumulation of its substrates, hypoxanthine and guanine, which are converted into uric acid by means of xanthine oxidase. Elevated APRT activity may also contribute to purine overproduction.

Several mechanisms can be identified that contribute to uric acid overproduction in HPRT deficiency [27,28]. a) HPRT catalyses the salvage synthesis of inosine monophosphate (IMP) and guanosine monophosphate (GMP) from the purine bases hypoxanthine and guanine respectively, utilizing 5'-phosphoribosyl-1-pyrophosphate (PRPP) as a co-substrate (Figure 1). The HPRT defect results in the accumulation of its substrates, hypoxanthine and guanine, which are converted into uric acid by means of xanthine oxidase. b) The increased availability of PRPP for PRPP amidotransferase, the rate-limiting enzyme of *de novo *synthesis of purine nucleotides (Figure 1), increases purine synthesis. c) On the other hand, there is decreased formation of PRPP amidotransferase feedback inhibitors, IMP and GMP. This dual mechanism results in an increased *de novo *synthesis of purine nucleotides. The combination of deficient recycling of purine bases with increased synthesis of purine nucleotides explains marked uric acid overproduction in HPRT deficiency. Elevated APRT activity may also contribute to purine overproduction.

### Pathophysiology of neurological symptoms

The pathophysiology of the neurological and behavioural dysfunctions remains unclear [29,30]. *Post mortem *studies of brains from Lesch-Nyhan patients have not disclosed any characteristic morphological abnormality [31,6]. Pioneer neurochemistry analysis of *post mortem *tissues revealed the first biochemical evidence of dysfunction of brain neurotransmitters in Lesch-Nyhan syndrome. In this study, biochemical aspects of the function of dopamine-neuron terminals in the striatum were decreased, whereas serotonin and 5-hydroxyindolacetic were increased [32]. Other biochemical studies of the Lesch Nyhan patient's cerebrospinal fluid showed a decreased level of the dopamine metabolite homivanillic acid, together with increased hypoxanthine and xanthine concentrations [31,33,34]. More recently, *in vivo *human studies with positron-emission tomography, in which ligands that bind to dopamine-related proteins in the brain have been used, have confirmed alterations of dopaminergic system in Lesch-Nyhan patients [35,36].

Two animal models, pharmacological and knockout, have been employed in the study of Lesch-Nyhan pathogenesis. The pharmacological rat model, developed by Breese *et al*., supports the relationship between self-injurious behaviour and dopamine deficit. Newborn animals treated with 6-hydroxydopamine, which destroys catecholamine-containing neurons, showed self-injurious behaviour in response to dopa agonist administration [37]. Unfortunately, HPRT-deficient knockout mice did not show neurological alterations, but they also presented an age-related decreased content of dopamine in their brains [38-40]. Various HPRT-deficient cell cultures have been developed to study the effects of the enzyme deficit and purine alterations [41,42] and dopamine deficit has been confirmed [43,44]. However, we must be careful when interpreting cell culture results since different HPRT-deficient cultures have showed different results [45]. Alterations in other neurotransmitters systems, such as serotonin [30] or adenosine neurotransmitters systems have also been implicated in patients with Lesch-Nyhan symptoms [46-49].

Among the biochemical alterations described in the central nervous system of Lesch-Nyhan patients, hypoxanthine excess is the most prominent. Toxic effects of this metabolite have been implicated in the pathogenesis of neurological dysfunction by means of alteration of adenosine transport [47,49], Na^+^K^+ ^ATPase activity [50], *etc*. Deficit of other purine compounds due to the enzyme defect is controversial, and altered nucleotide concentrations have been postulated as a possible cause of changes in G-protein-mediated signal transduction [51].

In summary, various studies have led to the suggestion that the neurological symptoms of Lesch-Nyhan syndrome could be related to a dysfunction of the dopaminergic neurotransmitter system in the basal ganglia, but the relationship between the dopamine deficit and the purine metabolic disorder is still unknown.

### Haematological aspects

Megaloblastic anaemia in Lesch-Nyhan patients is associated with megaloblastic findings in bone marrow and has been considered the result of increased folic acid consumption, due to enhanced *de novo *purine synthesis [52]. Nevertheless, this anaemia is not corrected by folate therapy.

## Diagnostic methods

The diagnosis of HPRT deficiency must be supported by clinical, biochemical, enzymatic and molecular data.

### Clinical and biochemical diagnosis

HPRT deficiency should be suspected in patients with hyperuricemia and uric acid overproduction with or without neurological impairment. During the first year of life, serum and urine uric acid determinations should be included in the differential diagnosis of psychomotor delay. Unfortunately, in previous years Lesch-Nyhan syndrome diagnosis has been delayed until self-mutilation was evident. Nephrolithiasis and obstructive nephropathy are common early manifestations in patients with partial HPRT deficiency.

Neuro imaging tests such as computing tomography (CT) and magnetic resonance imaging (MRI) may show atrophic non-specific changes [6,53]. Electroencephalograms are not diagnostic.

A high serum urate concentration is usually the biochemical finding that prompts special testing for the specific diagnosis, although some patients, particularly young infants, may have borderline serum uric acid levels due to increased renal clearance of uric acid [54]. Normal values for serum urate levels depend on age and sex. Similarly, the urinary uric acid/creatinine ratio can be employed as a screening test for inherited disorders of purine metabolism [54] but the values should be evaluated based on the age of the patient. Normal values for the urinary uric acid/creatinine ratio are below 1.0 after age 3-years. Mean plasma concentrations of urate, hypoxanthine, and xanthine, and their urinary excretion rates, are markedly elevated in HPRT-deficient patients. However, there is a non-statistically significant difference in these biochemical variables between partial and Lesch-Nyhan patients, except for xanthine urinary excretion that appears to be increased in Lesch-Nyhan patients as compared to partial HPRT deficient patients [55].

### Enzymatic diagnosis

HPRT deficiency must be confirmed by enzymatic determinations [56]. Patients present low or undetectable HPRT activity in haemolysates, with increased adenine phosphoribosyltransferase (APRT) activity. Lesch-Nyhan patients (grade 4) present undetectable HPRT activity, but in partial HPRT-deficient patients HPRT activity in haemolysate ranges from 0 to 10% [18,19]. Grade 1 patients usually show detectable haemolysate HPRT activity, while grade 2 or 3 patients generally do not. To better characterize the HPRT deficiency, enzyme activity can be measured in intact cells (erythrocytes or fibroblasts). A correlation was found between residual HPRT activity in intact erythrocytes and fibroblasts [19,57] and neurological involvement, although values may overlap for patients with very different phenotypes.

### Molecular diagnosis

Human HPRT is encoded by a single structural gene spanning approximately 45 Kb on the long arm of the X chromosome at Xq26, and consists of nine exons with a coding sequence of 654 bp [58,59]. Most HPRT-deficient patients present HPRT mRNA expression [60], and molecular diagnosis can be accomplished by cDNA sequencing [61]. In other cases, genomic DNA sequencing may be necessary [62]. Documented mutations in HPRT deficiency show a high degree of heterogeneity in type and location within the gene: deletions, insertions, duplications, and point mutations have been described as the cause of HPRT deficiency. To date, more than 300 disease-associated mutations have been found [63-65]. Single point mutations are the main cause of partial deficiency of the enzyme, whereas Lesch-Nyhan syndrome is caused mainly by mutations that modify the size of the predicted protein [64]. HPRT deficiency is inherited as an X-linked recessive trait. However, about 30% of patients' mothers are not somatic carriers, and these patients probably carry *de novo *mutations due to a germinal cell mutation. Molecular diagnosis in HPRT-deficient patients allows faster and more accurate carrier and prenatal diagnosis.

## Differential diagnosis

Complete HPRT deficiency-associated psychomotor delay must be differentiated from cerebral palsy. Self-injurious behaviour is also present in other conditions such as idiopathic mental retardation, autism, Tourette syndrome, Cornelia de Lange syndrome, severe psychiatric alterations, *etc*. In partial HPRT deficiency, differential diagnosis should explore other possible causes of hyperuricemia and gout. Elevated excretion of uric acid as an indication of purine overproduction is an important hallmark in the differential diagnosis. Other causes of hyperuricemia with purine overproduction include PRPP synthetase superactivity and glucose 6-phosphate deshydrogenase deficiency.

## Genetic counselling

Inheritance of HPRT deficiency is X-linked recessive. Thus males are generally affected and heterozygous female are carriers. However, at least five females with Lesch-Nyhan syndrome have been described, with different molecular alterations accounting for their HPRT deficiency [8,66-69].

Carrier diagnosis is an important issue for most HPRT-deficient families. Female carriers cannot be detected without the help of a laboratory since they are usually asymptomatic [70]. When the mutation is unknown, carrier status can be assessed by biochemical and enzymatic methods. Most female carriers for HPRT deficiency can be differentiated from non-carriers when 24-h urine samples are analysed after a 5-day purine-restricted diet: carriers have significantly higher urinary excretion rates of hypoxanthine and xanthine [71]. HPRT activity is most often normal in haemolysate of the peripheral blood of female carriers due to selection against HPRT-deficient erythrocyte precursors [72]. Enzymatic diagnosis of the carrier state can be performed by the identification of HPRT-deficient hair follicles or cultured fibroblasts [73] because of their mosaicism in terms of HPRT activity, although such diagnosis is not infallible. HPRT-deficient cells from carrier females can be selected based on their 6-thioguanine resistances. Proliferation assay of peripheral blood T-lymphocytes in the presence of 6-thioguanine is diagnostic in most cases [74].

When the HPRT mutation has been characterized in the family, faster and more accurate carrier diagnosis can be performed by molecular methods [75].

## Antenatal diagnosis

Prenatal diagnosis for Lesch-Nyhan syndrome can be performed with amniotic cells obtained by amniocentesis at about 15–18 weeks' gestation, or chorionic villus cells obtained at about 10–12 weeks' gestation. Both HPRT enzymatic assay and molecular analysis for the known disease-causing mutation can be performed [76,77].

## Treatment

### Uric acid overproduction

Uric acid overproduction can be controlled with the xanthine oxidase inhibitor allopurinol that blocks the conversion of xanthine and hypoxanthine into uric acid [78]. Allopurinol treatment reduces serum urate and urine uric acid levels and thus prevents uric acid crystalluria, nephrolithiasis, gouty arthritis and tophi [4,55,79,80]. Allopurinol should be started as soon as the enzyme deficiency has been diagnosed, although it has no effect on behavioural and neurological symptoms. In adults, combined treatment with colchicine prophylaxis may be required to prevent acute inflammation. In our experience [55], treatment with allopurinol normalized serum urate level in all patients and resulted in a mean reduction of serum urate of about 50% and a 74% reduction in urinary uric acid/creatinine ratio. In contrast, allopurinol treatment increased mean hypoxanthine and xanthine urinary excretion rates about 5- and 10-fold, respectively, compared to baseline levels. Allopurinol-related biochemical changes are similar in patients with either complete or partial HPRT deficiency. Renal function usually remained stable or improved with treatment. Allopurinol doses ranged from 50 to 600 mg/day. The initial dosage of allopurinol is 5–10 mg/Kg/day and it should be adjusted to maintain high-normal serum uric acid levels and a urinary uric acid/creatinine ratio lower than 1.0. Allopurinol is efficacious and generally safe for the treatment of uric acid overproduction in patients with HPRT deficiency [55]. However, xanthine lithiasis may develop as a consequence of allopurinol therapy [81]. The optimal allopurinol dose for HPRT-deficient patients has not been established. In our experience, when serum urate is maintained close to its solubility threshold, urate deposition does not occur. Xanthine lithiasis may be prevented by sequential determination of urinary oxypurines, which should be at a certain balance with uric acid excretion.

### Motor syndrome

The lack of precise understanding of the cause of the neurological dysfunction has precluded the development of useful therapies. Spasticity and dystonia can be managed with benzodiazepines and gamma-aminobutyric acid inhibitors such as baclofen [6]. No medication has been found to effectively control the extrapyramidal manifestations of the disease. Physical rehabilitation, including management of dysarthria and dysphagia, special devices to enable hand control of objects, appropriated walking aids, and a programme of posture management to prevent deformities is recommended.

### Behavioural manifestations

**Figure 2:**
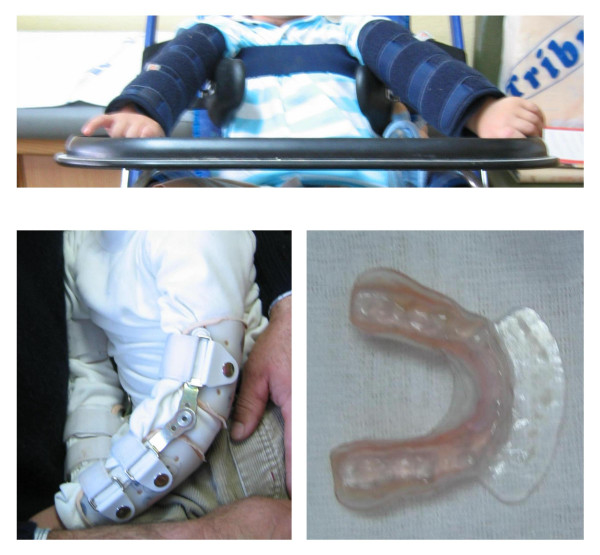
**Management of self-injurious behaviour**. The cornerstone of day-to-day management of Lesch-Nyhan syndrome is still adapted physical restraint to protect patients from themselves. For instance, elbow restraints allow hand use without the possibility of finger mutilation, and dental guards prevent cheek biting. Patients themselves request restrictions and became anxious if they are unrestrained.

Self-injurious behaviour must be managed with a combination of physical restraints, behavioural and pharmaceutical treatments [82]. Benzodiazepines and carbamazapine are sometimes useful for ameliorating behavioural manifestations and anxiety. Stress increases self-injurious behaviour. Thus, stressful situations should be avoided and aversive techniques should not be employed. Instead, behavioural extinction methods have proven to be partially efficacious in a controlled setting. However, the cornerstone of day-to-day management of Lesch-Nyhan syndrome is still adapted physical restraint to protect patients from themselves. For instance, elbow restraints allow hand use without the possibility of finger mutilation, and dental guards prevent cheek biting (Figure 2). Patients themselves request restrictions and became anxious if they are unrestrained, and sometimes restraints that would appear to be ineffective, such as a pair of gloves to prevent finger biting, are useful.

### Therapies under investigation

Some reports have suggested that gabapentin may improve self-injurious behaviour and no side effects have been associated with its use [83]. Other treatments under investigation for the management of self-injurious behaviour are local injections of botulin toxin [84], deep brain stimulation in globus pallidus [85,86] or dopamine replacement therapy, but they need to prove to be efficacious and safe in the long-term management of these patients.

## Prognosis

With appropriate allopurinol treatment, renal function is generally preserved [55] and patients survive until second or third decade of life. Patients with the complete Lesch Nyhan syndrome are unable to walk and are confined to a wheelchair. Generally with restriction and medical treatment self-injurious behaviour can be managed appropriately. Causes of death include pneumonia and other infectious diseases. In some cases sudden and unexpected death has been reported. This appears to have a respiratory rather than a cardiogenic origin [87].
